# Diagnostic Performance of T2Candida Among ICU Patients With Risk Factors for Invasive Candidiasis

**DOI:** 10.1093/ofid/ofz136

**Published:** 2019-03-25

**Authors:** Maiken Cavling Arendrup, Jakob S Andersen, Mads Kristian Holten, Kenneth B Krarup, Nanna Reiter, Jens Schierbeck, Marie Helleberg

**Affiliations:** 1 Unit of Mycology, Statens Serum Institut, Copenhagen, Denmark; 2 Department of Clinical Microbiology, Copenhagen University Hospital Rigshospitalet, Copenhagen, Denmark; 3 Department of Clinical Medicine, University of Copenhagen, Copenhagen, Denmark; 4 Department of Intensive Care Medicine, Copenhagen University Hospital Rigshospitalet, Copenhagen, Denmark; 5 Department of Intensive Care Medicine, Odense University Hospital, Odense, Denmark; 6 Department of Infectious Diseases, Copenhagen University Hospital Rigshospitalet, Copenhagen, Denmark

**Keywords:** blood culture, *Candida*, candida mannan antigen, diagnostic performance, intensive care unit

## Abstract

**Background:**

Invasive candidiasis (IC) comprises candidemia and deep-seated candidiasis. Blood culture (BC) is the gold standard test, but sensitivity is low. T2Candida is a new diagnostic test. We investigated the performance of T2Candida, BC, and *Candida* mannan antigen (MAg) for detection of IC in a high-risk intensive care unit (ICU) population.

**Methods:**

One-hundred twenty-six ICU patients at high risk of IC with sepsis despite 3 days of broad-spectrum antibiotics were included. Paired BC, T2Candida, and MAg were obtained twice weekly (334 sets). Patients were classified into proven, likely, possible, or unlikely IC based on patient record review.

**Results:**

At enrollment, 92 (77%) patients were receiving antifungal therapy (mainly fluconazole 66%). Fifteen (11.9%) patients were positive by BC (n = 4), T2Candida (n = 11), or MAg (n = 10). The T2Candida species distribution at inclusion (*Candida albicans*/*Candida tropicalis*: 8/11 [72.3%] and *Candida glabrata*/*Candida krusei*: 3/11 [27.3%]) was supported by the identification of BC or colonizing isolates in 10/11 cases. Patients were classified with proven (11), likely (6), possible (11), and unlikely (98) IC. Defining IC as proven/proven&likely/proven&likely&possible, respectively, the sensitivity was as follows: T2Candida (55%/59%/39%), BC (45%/29%/ 8%), and MAg (36%/41%/32%). The negative predictive value was similar across the tests for proven vs others and proven/likely vs others (94%–96% and 90%–95%, respectively). For test combinations including T2Candida, the sensitivity increased to 64%–65%, without hampering the positive predictive value.

**Conclusions:**

In conclusion, although the diagnostic performance was modest for all the tests, the combination of T2Candida and BC seemed to have the best diagnostic performance, and thus implementation of T2Candida may improve the diagnosis of IC.

Invasive candidiasis (IC) comprises candidemia and deep-seated candidiasis with or without concomitant candidemia [1]. The most important risk groups are patients in the intensive care unit (ICU) and those undergoing abdominal surgery, particularly if repeated or complicated [2–6]. Candidemia is associated with significant morbidity, mortality, and cost [1]. The overall mortality is around 40% [7] but is higher among patients in the ICU [8].

A positive blood culture (BC) or culture from a newly (<24 hours) placed drain establishes the diagnosis of proven IC [9]. However, BCs have a low sensitivity. Autopsy studies have suggested a sensitivity between 21% and 71%, which is highest if large-volume BCs (60 mL) are obtained daily and additional sets are obtained during febrile episodes [10]. The use of mycosis culture bottles improves sensitivity for the detection of *Candida glabrata* when the BACTEC system is used and of polymicrobial infections when the BacT/ALERT system is used [2, 11–13]. However, such bottles are infrequently used.

The time to BC positivity is around 2 days [2, 14]. A 2-day delay in antifungal therapy has been associated with more than a doubling of the mortality rate in several [15, 16] but not all studies [2, 17, 18]. Hence, rapid diagnostic tests are warranted. β-D-glucan and the *Candida* mannan antigen (MAg) and antibody (MAb) tests are recommended biomarkers for the diagnosis and management of *Candida* diseases [19]. The β-D-glucan is a panfungal test and hence cannot distinguish between candidiasis and other fungal infections. The sensitivity for IC is generally high (76.7%–100.0%), but the specificity is more variable (40.0%–91.8%) [20–25]. Multiple sources for false positivity have been reported, many of which are particularly challenging in the ICU [20, 21, 26]. This is an important caveat and may lead to inappropriate antifungal treatment of patients without candidiasis. *Candida* MAg and MAb detection has been found useful for the detection of IC [27–31], although the performance of combined MAg and MAb detection was disappointing (sensitivity 55% and specificity 60%) in a recent study including ICU patients with severe abdominal conditions [20]. The variation in reported performance for this test remains unexplained, but in a population mainly at risk of candidiasis, a *Candida*-specific rather than panfungal test may be preferable with respect to risk of false-positive results and interpretation.

The T2Candida molecular test has recently been Conformité Européenne (CE) marked and Food and Drug Administration (FDA) cleared. The test detects (1) *C. albicans* and *C. tropicalis,* reported together as *C. albicans*/*C. tropicalis*; (2) *C. glabrata*, *C. krusei*, *S. cerevisiae,* and *C. bracarensis*, reported together as *C. glabrata*/*C. krusei*; and (3) *C. parapsilosis, C. orthopsilosis,* and *C. metapsilosis*, reported as *C. parapsilosis*. Together, these species formed the majority (93.5%) of the *Candida* bloodstream infections in Denmark in 2012–2015 [11]. The combined sensitivity and specificity of the test for detecting candidemia were 91.1% and 99.4%, respectively, in the study that led to the FDA clearance [32]. Subsequent studies have confirmed a high sensitivity using seeded blood samples [33] and when comparing the results for follow-up BCs and T2Candida tests obtained from pediatric and adult patients with documented candidemia [14, 34]. Moreover, among patients already receiving antifungal therapy, the T2Candida assay yielded positive results in a number of cases with negative BC [14]. Of note, studies performed so far included candidemic patients with or without enrichment of the study material with spiked blood samples. Hence the performance in cases with IC without concomitant candidemia is largely unknown.

In this study, we compared the diagnostic performance of BC, T2Candida, and *Candida* MAg and MAb in patients at risk of IC in the 2 major university ICUs in a country with a high incidence of IC [11, 35]. To our knowledge, it is the largest and only non-single-center prospective study performed in a high-risk ICU setting for comparative diagnosis of IC.

## METHODS

**Patients and Data Collection**

In total, 126 ICU patients were included during the study period of October 1, 2014, to June 21, 2016. The characteristics at 2 major university ICU units are summarized in Supplementary Table 1.

**Inclusion Criterion**

The inclusion criterion was 1 of the following: (1) initiation of prophylactic, empiric, or preemptive antifungal treatment; (2) colonization index ≥0.5; or 3) temperature >38°C or <36°C despite 3 days of broad-spectrum antibiotic treatment *and* at least 2 of the following risk factors: abdominal surgery, secondary peritonitis, pancreatitis, central vein catheter (CVC) in place, total parenteral nutrition (TPN), dialysis, steroid treatment, immunosuppressive treatment, or liver transplantation. Patients who fulfilled the inclusion criterion but had candidemia before inclusion were not excluded. Presence or absence of each inclusion criterion was registered, along with each blood sampling. Upon completion of the study, patient records and clinical microbiology test databases were reviewed for each patient. The following data were extracted: underlying diseases, body temperature >38°C or <36°C, signs of sepsis (systolic blood pressure <90 mmHg; mean arterial pressure <65 mmHg; need of vasopressor infusion [epi-/norepinephrine] or blood pressure drop 40 mmHg from baseline), white blood cell count >12 000 cells/µL, confirmation of ≥3 days of broad-spectrum antibiotics. The microbiological tests were reviewed and categorized into whether *Candida* were isolated from at least 2 nonsterile sites (±3 days) and whether there was an alternative microbiological diagnosis.

From each patient, a 5.5-mL EDTA tube and a BC set were obtained simultaneously at the time of enrollment. The EDTA tube was used for T2Candida (T2 Biosystems, Lexington, MA) and for Platelia *Candida* MAg and Ab (Bio-Rad, Marnes-la-Coquette, France). BacT/ALERT (bioMérieux, Marcy l’Etoile, France) BC was used in Odense and BACTEC (Becton Dickinson, Franklin Lakes, NJ) at Rigshospitalet. Sampling was repeated twice weekly in Odense for as long as the patient fulfilled the enrollment criteria. At Rigshospitalet, a single follow-up sample set was obtained after 3 days. At both hospitals, routine urine and tracheal secretions were cultured at regular intervals. Additional microbiological tests were performed as indicated by the treating physician.

Cases were classified based on vital signs and results of microbiological examinations within 3 days of enrollment (see below).

Proven IC:  either (1) growth of *Candida* in a BC or (2) fulfilling all the following: (i) growth of *Candida* in a tissue biopsy or sample from a drain placed within 24 hours, (ii) sampling from a normally sterile site using sterile procedures, and (iii) clinical or radiological signs of infection at that site (EORTC/MSG criteria [9]).
Likely IC:  either (1) *Candida* isolated from at least 2 nonsterile sites (±3 days) and no alternative microbiological diagnosis (±3 days) and fulfilling ≥1 SIRS criterion despite 3 days of broad-spectrum antibacterial treatment or (2) *Candida* MAg >250 mg/L and colonized with *Candida* at 2 sites (±3 days) (adopted from [25, 36]).
Possible IC: either (1) *Candida* MAg >125 mg/L and colonized at 2 sites (±3 days) or (2) *Candida* MAg >250 mg/L or 3) *Candida* isolated from at least 2 nonsterile sites (±3 days) and fulfilling SIRS criteria despite ≥3 days of broad-spectrum antibiotics.
No IC: remaining cases.

Subsequently, we studied the medical files of all cases who were initially classified as either proven, likely, or possible IC or who had at least 1 positive marker for IC (T2 or mannan Ag) and revised the classification according to results of microbiological analyses within 21 days of enrollment and detailed information on clinical course, as presented in Supplementary Table 2.

### Statistical Analysis

The sensitivity, specificity, and positive (PPV) and negative (NPV) predictive values of the tests were assessed based on the sample drawn at inclusion and the subsequent sample drawn at day 2 or 3. If analyses of the 2 samples were discordant (ie, 1 positive and 1 negative), the test was considered positive. The Fisher exact test was used to compare the proportions of patients receiving antifungal therapy for BC-positive vs -negative patients.

## RESULTS

In total, 334 sample sets from 126 ICU patients at risk for IC were included during the 21-month study period. The median age (range) was 65.5 (16–89) years, and 72 (57%) were men. The majority of patients met more than 1 inclusion criterion (82%) and received antifungal therapy at the time of inclusion (77%) (Table 1). Abdominal surgery was the most common risk factor (44%), followed by secondary peritonitis or immunosuppression.

**Table 1.** Baseline Characteristics of the Study Population (126 Patients^a^)

Male, No. (%)	72 (57)
Age, median (IQR), y	65.5 (53–75)
Inclusion criterion, %	
Initiation of antifungal therapy	84
Colonization index ≥ 0.5	33
Fever despite antibiotics combined with the presence of at least 2 risk factors	80
Risk factors, No. (%)	
Central venous catheter	112/124 (90)
Parenteral nutrition	24/121 (20)
Dialysis	26/122 (21)
Steroids	32/121 (26)
Abdominal surgery	54/123 (44)
Secondary peritonitis	20/120 (17)
Pancreatitis	6/120 (5)
Immunosuppressed	19/121 (16)
Liver transplantation	1/121 (0.8)
Antifungal Rx at time of inclusion, No. (%)	
None	27/119 (23)
Fluconazole^b^	76/119 (63)
Other azole	3/119 (3)
Echinocandin^b^	14/119 (12)

Abbreviation: IQR, interquartile range.

^a^Data missing for some patients as indicated by varying denominators.

^b^One patient received caspofungin and fluconazole combination therapy.

### Microbiology

**Figure 1.**
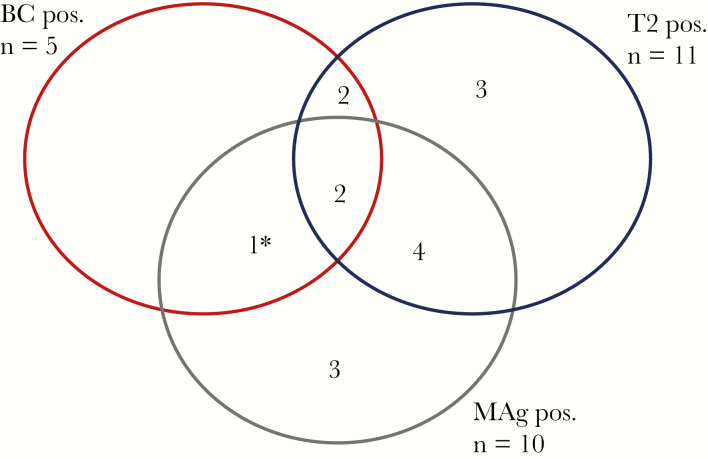
Positive diagnostic samples at the time of inclusion in the study. ^a^Blood culture with *Candida kefyr,* which is not included in the panel of the T2Candida test. Abbreviations: BC, blood culture; MAg, mannan antigen; Pos., positive; T2, T2Candida.

At the time of enrollment, 15 (11.9%) patients were positive by BC, T2Candida, and/or MAg testing (Figure 1, Table 2). Overall, 7/11 T2Candida-positive samples and 7/10 MAg-positive samples were from patients without a concomitant positive BC at the time of inclusion. Significantly fewer patients with positive compared with negative BCs received antifungal therapy at the time of inclusion (2/5 [40%] vs 90/115 [78%], *P* = .008). For the T2Candida test and the MAg test, there were no statistically significant associations between test results and antifungal therapy at inclusion (7/11 [64%] vs 80/103 [78%], *P* = .287, and 9/10 [90%] vs 78/103 [76%], *P* = .449, respectively).

**Table 2.** Comparison of Blood Culture, T2Candida, and *Candida* Mannan Antigen Results for the Initial Samples Taken at the Time of Inclusion

Blood Culture	T2Candida				*Candida* MAg			T2Candida and/or MAg			
	*Candida albicans/tropicalis*	*Candida glabrata/krusei*	Neg.	Invalid	Pos.	Int.	Neg.	+/+	+/−	−/+	−/−
*C. albicans* (1)	1						1		1		
*C. glabrata* (1)		1					1		1		
*C. tropicalis* (1)	1				1			1			
*C. tropicalis & C. glabrata* (1)	1		-^a^		1			1			
*C. kefyr* (1)			1		1					1	
Neg-BC (121)	5	2	107	7	7	7	107	4	3	3	111
In total (126)	8	3	108	7	10	7	109	6	5	4	111

Abbreviations: Int., intermediate; MAg, mannan antigen; Neg., negative; Neg-BC, blood culture without growth of *Candida*; Pos., positive.

^a^The *C. glabrata* found in the initial blood culture was reported on day 4 but not on day 0 by the T2Candida test.

### T2Candida Test Results

The species distribution among patients with initial samples positive in T2Candida was *C. albicans*/*C. tropicalis* in 8/11 (72.3%) patients and *C. glabrata*/*C. krusei* in 3/11 (27.3%). The T2Candida test was positive for 4/5 patients with positive index BCs and for 2 additional patients with positive BCs 2 and 4 days before inclusion, whereas 5 patients had negative BCs (Table 3). The species group reported by the T2Candida test was confirmed by the identification of BC or colonizing isolates in 10/11 cases (*Candida* was not identified to the species level in 1 patient), although T2Candida failed to detect the second species at day 0 in a patient with polyfungal infection (Table 3). T2Candida was negative in the patient with *C. kefyr* candidemia, a species that is not included in the panel, and was invalid in 9/126 (7.1%) patients at enrollment.

**Table 3.** Kinetics of the Blood Culture and Biomarker Results

Classification at Inclusion, Pt No.	Test	Days From Study Enrollment										
		0	1–2	3–4	5–6	7–9	10–12	13–14	15–16	19–20	21	23
Blood culture–pos. cases												
Proven, #63	BC	T & G		Neg								
	T2	A/T		A/T & G/K								
	MAg	>500		>500								
	MAb	Int		Int								
Proven, #103	BC	T	T	Neg	T	Neg	Neg	Neg	Neg	Neg		
	T2	A/T	A/T	A/T	Neg	A/T	Neg	Neg	Neg	Neg		
	MAg	>500	>500	>500	>500	>500	>500	>500	>500	>500		
	MAb	Neg	Neg	Neg	15.9	37.2	38.2	37.2	35.7	38.6		
Proven, #4	BC	G		Neg								
	T2	G/K		Neg								
	MAg	Neg		Neg								
	MAb	Int		Int								
Proven, #98	BC	A		Neg								
	T2	A/T		A/T								
	MAg	Neg		Neg								
	MAb	Neg		Neg								
Proven, #33	BC	*C. kefyr*										
	T2	Neg										
	MAg	>500										
	MAb	Int										
T2Candida+, BC-neg. cases												
Proven (*C. albicans*), #89	T2	A/T		A/T								
	MAg	Neg		Int								
	MAb	14.2		11.7								
Likely (*C. krusei*), #60	BC	Neg		Neg	*C. dubliniensis*	Neg						
	T2	G/K		Neg	P	Neg						
	MAg	>500		>500	>500	>500						
	MAb	15.1		13.2	19.8	32.6						
Likely (*C. tropicalis*), #22	T2	A/T		A/T								
	MAg	>500		>500								
	MAb	Int		35.3								
Likely (*C. albicans*), #28	T2	A/T		A/T								
	MAg	>500		>500								
	MAb	Neg		Neg								
Proven (*Candida* no ID), #90	T2	G/K		Neg	Neg							
	MAg	>500		>500	250–500							
	MAb	Neg		Neg	Neg							
Likely (prov d-4, *C. albicans*), #54	T2	A/T										
	MAg	Neg										
	MAb	Int										
Possible *C. albicans*, #114	T2	A/T	Invalid	A/T	A/T		A/T	A/T	Neg			
	MAg	Neg	Neg	Neg	Neg		Neg	Neg	Neg			
	MAb	Int	Int	Int	37.2		58.6	58.6	58.6			
Possible *C. albicans*, #74	T2	Neg	Neg	G/K								
	MAg	Neg	Neg	125–250								
	MAb	Neg	Neg	10.4								
Unlikely, #36	T2	Neg	Neg		Neg		Neg	Neg	Neg	Neg	P	Neg
	MAg	Neg	Int		125.2		Neg	Neg	Neg	Neg	Neg	Int
	MAb	20.9	24.7		45.2		38.9	37.8	41.1	39.8	35.2	40.8
Unlikely, #83	T2	Neg	P		Neg	Neg	Neg					
	MAg	Neg	Neg		Neg	Neg	Neg					
	MAb	Neg	Neg		Neg	Neg	Neg					
Mannan AG–pos., T2C- and BC-neg. cases												
Unlikely, #109	T2	Neg										
	MAg	>500										
	MAb	Neg										
Unlikely (prov d-5,-7, *C. albicans*), #20	T2	Neg										
	MAg	>500										
	MAb	71.1										
Unlikely, #24	T2	Neg	Neg	Neg	Neg	Neg						
	MAg	125–250	125–250	125–250	125–250	>250						
	MAb	71.7	65.9	75	54.7	75.4						
Possible (Prov d+4), #121	T2	Neg	Neg	Neg	Neg	Neg						
	MAg	Neg	Neg	Neg	Neg	125–250						
	MAb	Int	11.2	14.6	19.2	34.7						
Unlikely, #70	T2	Neg	Neg	Neg								
	MAg	Neg	125–250	125–250								
	MAb	Neg	Neg	Neg								

Empty space indicates that no samples were obtained in that time period. For patients with no BC row included, the accompanying blood cultures were negative. Patient 20 was blood culture positive with *C. albicans* 5 and 7 days before enrollment in the study. Mannan antigen interpretative cutoffs: Neg.: <62.5 pg/mL; Int.: ≥62.5–125 pg/mL; and Pos.: ≥125 pg/mL. Antimannan antibody interpretative cutoffs: Neg.: <5 AU/mL; Int.: 5–<10 AU/mL; and Pos.: ≥10 AU/mL.

Abbreviations: BC, blood culture; Int, intermediate; MAb, mannan antibody (AU/mL); MAg, mannan antigen (pg/mL); Neg, negative; T2, T2Candida.

Fungal species abbreviations: A, *Candida albicans*; G, *Candida glabrata*; K, *Candida krusei*; P, *Candida parapsilosis*; T, *Candida tropicalis.*

In 4 BC-negative patients, a single follow-up sample was positive by T2Candida, 3 of these with *C. parapsilosis* in the absence of concomitant *C. parapsilosis* colonization or other indication of IC (Table 3). These 3 cases were likely false positives. The fourth patient became positive with *C. glabrata*/*C. krusei*, MAg, and MAb at day 4. Including these, the overall species distribution as determined by T2Candida was *C. albicans*/*C. tropicalis* 8/15 (53%), *C. glabrata*/*C. krusei* 4/15 (27%), and *C. parapsilosis* 3/15 (20%).

### *Candida* Mannan Ag and Ab

The MAg test was positive in 3/5 patients with positive BC at enrollment, including the patient with *C. kefyr* candidemia (Table 3). Seven additional patients were MAg positive at enrollment, 4 of whom were T2Candida positive and 1 of whom had been BC positive 5 and 7 days earlier. During the follow-up period, 4 additional BC-negative patients changed to MAg positive (2 of whom also developed MAb), whereas 5 patients became MAb positive during follow-up (1 of whom was BC positive at inclusion, 4 of whom were T2 positive, and 4 of whom were or subsequently became MAg positive).

### Kinetics of BC and Biomarker Results

T2Candida stayed positive longer (mean [range], 3.2 [0–5] days) than BC. Eight of 9 patients with positive MAg and follow-up samples remained MAg positive during the entire observation period (up to 20 days) (Table 3).

### Clinical Classification and Performance of Diagnostic Tests

The numbers of patients classified as proven, likely, or possible IC were 11 (8.7%), 6 (4.8%), and 11 (8.7%), respectively (Table 4; Supplementary Table 2). Abdominal IC was the most common manifestation (16/22 [72.7%]) among BC-negative patients with proven (2/5), likely (4/6), or possible IC (10/11), respectively. Significantly fewer patients with proven IC received antifungal treatment (AF) before enrollment (5/11) than among likely and unlikely cases (6/6, *P* = .004, and 82/95, *P* = .047) (Table 4).

**Table 4.** Performance of Blood Culture, T2Candida, and Mannan Antigen Based Upon the 2 Initial Blood Sample Sets for Patients Classified With Proven (11), Likely (6), Possible (11), or Unlikely (98) Invasive Candidiasis

Final Classification	No. of Patients	No. w/ Prior AF	BC Pos	T2 Pos	MAg Pos
Proven	11	5/11	5/11	6/11	4/11
Candidemia within ±3 d of inclusion	6	2^a,a^	5/6	5/6	3/6
Abdominal candidiasis incl. aortaprothesis	1	0	0	0	0
Abdominal candidiasis	1	1^e^	0	1/1	1/1
Mediastinal or pleural candidiasis	2	2^b,e^	0	0	0
Polymicrobial necrotizing fasciitis	1	0	0	0	0
Likely	6	6/6	0/6	4/6	3/6
Abdominal candidiasis & prior proven abdominal candidiasis	3	3^a,d,e^	0	1/3	0
Abdominal candidiasis	1	1^c^	0	1/1	1/1
Pulmonary candidiasis in hematological Pt	1	1^e^	0	1/1	1/1
Tissue candidiasis	1	1^a^	0	1/1	1/1
Possible	11	10/11	0/11	1 (&1 d4)/11	2/11
Abdominal candidiasis	8	7^a,a,b,b,b,e,e^	0	1 (&1 d4)/8	1/8
Abdominal candidiasis & prior proven candidemia, d –5 & –7	1	1^c^	0	0	1/8
Abdominal candidiasis & proven, d +4	1	1^e^	0	0	0
Renal candidiasis & prior proven	1	1^e^	0	0	0
Unlikely	98	82/95^f^	0/98	1^g^ /98	2/98

Information on prior systemic antifungal therapy at inclusion is indicated (No. w/ Prior AF).

Abbreviations: AF, antifungal treatment; BC, blood culture; MAg, mannan antigen (pg/mL); T2, T2Candida.

^a^Duration of AF before inclusion: <1 day.

^b^Duration of AF before inclusion: 1–3 days.

^c^Duration of AF before inclusion: 4–7 days.

^d^Duration of AF before inclusion: >7 days.

^e^No information on duration of AF before inclusion.

^f^Information on prior antifungal therapy was missing for 3/98 patients.

^g^ *Candida parapsilosis*.

The sensitivity was higher for T2Candida compared with BC and MAg for proven IC (55% vs 45% and 36%), for proven or likely IC (59% vs 29% and 41%), and for proven, likely, or possible IC (39% vs 8% and 32%), respectively, compared with others (Table 5); however, as the number of cases of IC was limited, these differences did not reach statistical significance. When combining the diagnostic tests, the sensitivity increased to 64%–65% for test combinations including T2Candida compared with 53%–55% for BC+MAg for proven vs others and proven/likely vs others. The specificity was high and above 90% for all tests and test combinations except T2Candida-BC-MAg triple testing for proven vs others. The PPV was higher for T2Candida than for MAg (50% vs 36% for proven and 83% vs 64% for proven/likely vs other, respectively). The negative predictive value was similar across the tests (94%–96% for proven and 90%–95% for proven/likely, respectively) but somewhat lower if including possible cases of IC (81%–88%). Overall, T2Candida combined with BC seemed to have the best diagnostic performance for proven/likely IC compared with other single tests or test combinations.

**Table 5.** Performance Characteristics for the Diagnostic Tests Using the Clinical Classification of Proven (11), Likely (6), Possible (11), or Unlikely Invasive Candidiasis (98) as the Gold Standard

Candidiasis Classification and Diagnostic Test	Sensitivity, %	Specificity, %	PPV, %	NPV, %
Proven vs others				
BC	45	100	100	95
T2	55	93	50	96
MAg	36	94	36	94
T2+BC	64	93	54	96
T2+MAg	64	89	41	96
BC+MAg	55	94	38	96
T2+BC+MAg	64	89	41	96
Proven or likely vs others				
BC	29	100	100	90
T2	59	96	83	94
MAg	41	96	64	91
T2+BC	65	96	85	95
T2+MAg	65	92	65	94
BC+MAg	53	96	54	93
T2+BC+MAg	65	92	65	94
Proven, likely, or possible vs unlikely				
BC	18	100	100	81
T2	39	97	92	85
MAg	32	98	82	83
T2+BC	43	97	92	86
T2+MAg	50	95	82	88
BC+MAg	39	98	85	85
T2+BC+MAg	50	98	82	88

Abbreviations: BC, blood culture; Mag, mannan antigen; NPV, negative predictive value; PPV, positive predictive value; T2, T2Candida.

## Discussion

In this study comparing the performance of diagnostic tests among high-risk ICU patients, the highest sensitivity and NPV for IC were found for T2Candida and for test combinations including T2Candida. No single test or test combination resulted in a sensitivity above 65% for IC, but the combination of T2Candida and BC seemed to have the best diagnostic performance for proven/likely IC. So far, the only other study prospectively enrolling patients before the establishment of an IC diagnosis found the T2Candida test positive in 4/6 patients with positive BCs, in line with the 4/5 found in our study [32]. In that study, a 91.1% sensitivity and 99.4% specificity for T2Candida compared with BC were estimated, enriching the sample size with 250 samples spiked with *Candida* cells at concentrations similar to what is expected during (untreated) candidemia [32]. We estimated test performance based on the clinical classification of high-risk patients, the majority of whom were suffering from deep-seated IC. We speculate that this difference in study design and the fact that many patients had received several days of antifungal therapy before enrollment may in part explain the lower performance of the T2Candida test in our setting.

The expected IC rate is around 10% among severely ill high-risk ICU patients [37]. In this study, 8.7% were classified with proven IC and additional 4.8% with likely IC. In this context, the rate of BC-proven candidiasis was notably low (4%). Most of our patients received antifungal therapy at the time of enrollment, with fluconazole being the predominating compound. Notably, none of the patients with candidemia had received AF for ≥24 hours at the time of inclusion. Fluconazole (20 mg/L) reduced overall BC sensitivity by 7.5%–12.5% in a recent laboratory experiment [38]. In line with these findings, significantly fewer patients with proven infection received prior antifungal therapy in our study, a diagnosis that requires a positive culture from blood or a sterile site. In contrast, fluconazole was observed not to impact the sensitivity of the T2Candida assay in the above-mentioned laboratory study [38]. We observed a trend toward a lower T2Candida-positive rate for patients receiving antifungal therapy before sampling, suggesting that T2Candida sensitivity may also be reduced during antifungal therapy. Fluconazole therapy may lower the fungal load or clear the bloodstream despite not clearing the foci of patients with deep-seated candidiasis and hence result in a lower T2Candida sensitivity that is not observed in studies of spiked blood. It may therefore be advisable to initiate diagnostic testing before initiation of antifungals whenever feasible.

The species group provided for positive T2Candida test results was in agreement with the *Candida* species diagnosis established by culture from blood or other sites for patients with IC. The overall T2Candida species distribution including positives during follow-up was *C. albicans*/*C. tropicalis* 53%, *C. glabrata*/*C. krusei* 27%, and *C. parapsilosis* 20%. In comparison, these species accounted for 52%, 36%, and 3%, respectively, in the national surveillance program in 2012–2015 [11]. *C. parapsilosis* is a skin and biofilm–associated organism. None of the 3 patients with a positive *C. parapsilosis* T2Candida result were diagnosed as IC or colonized with *C. parapsilosis* by culture. We therefore regard these findings as contaminations, illustrating the importance of thoroughly decontaminating the skin or catheter before blood draws for T2Candida. In settings with a higher true prevalence of *C. parapsilosis* infections like in neonates and in endemic areas such as Latin America, Southern Europe, and Asia, it may be difficult to differentiate clinically relevant *C. parapsilosis* findings from contaminations, potentially leading to unnecessary use of antifungal therapy [39].

T2Candida is an expensive diagnostic tool. At the time of writing, the price in our country for a test kit is US$300 per sample, and an annual fee for a service contract of US$15 500. Although cost analyses have suggested lower candidemia-related inpatient costs and mortality with implementation of the T2Candida test [40], cost may be prohibitive in some settings, particularly at a time of declining cost for the echinocandins. The combination of MAg with BC was superior to BC alone. However, in addition to being less sensitive compared with T2Candida alone and when combined with BC, a drawback is that species identification is not provided for MAg-positive samples to inform whether the species is a likely target for de-escalation to fluconazole once the patient is stable.

The time to negativity was longer for the T2Candida test than for BC, supporting recent findings [41]. This may suggest that whereas the appropriate duration of therapy after resolution of symptoms and BC negativity is 14 days, this may be shorter after resolution of symptoms and T2Candida negativity [42]. In contrast, the mannan antigen remained positive throughout the observation period and up to 14 days after BC documented clearance of the bloodstream and thus should not alone prompt continued antifungal therapy.

This study has limitations. First and foremost, the number of proven and likely cases was limited, and more than half of the proven IC cases were BC negative at the time of inclusion, suggesting a low amount of circulating *Candida* cells. Therefore, numbers were insufficient for statistical comparative analysis of the diagnostic tests. In the absence of a reliable gold standard for the diagnosis of IC, classification of patients was done adopting the EORTC/MSG definitions for proven infection and criteria adopted from 2 recent studies for likely and possible IC [9, 25, 36]. A positive blood culture defined proven infection, and a highly positive MAg in a colonized patient with clinical signs and symptoms defined likely IC, whereas a positive T2Candida did not. Consequently, the relative diagnostic performance of T2Candida compared with BC and MAg may have been underestimated. Most of the patients with deep-seated IC were patients with complicated infections receiving broad-spectrum antibiotics in whom it is difficult to dissect the clinical significance of the findings of *Candida* in microbiological samples. Finally, colonization was difficult to compare, as neither sampling nor mycologic examination was standardized.

In conclusion, our study underscores the complexity associated with diagnosing IC. Most patients were BC negative, probably in part because the majority were already on fluconazole prophylaxis, as recommended in a multimorbid high-risk population. T2Candida was the biomarker that contributed the most to improving the diagnostic sensitivity while retaining a good specificity and NPV. This makes it a promising addition to the diagnostic armamentarium. However, our study also suggests that maximal benefit of diagnostic efforts may require that sampling is initiated as early as possible, preferably before initiation of antifungal therapy, when the sensitivity and NPV of T2Candida and BC testing are maximal and probably sufficient for excluding IC.

## Supplementary Data

Supplementary materials are available at *Open Forum Infectious Diseases* online. Consisting of data provided by the authors to benefit the reader, the posted materials are not copyedited and are the sole responsibility of the authors, so questions or comments should be addressed to the corresponding author.

ofz136_suppl_supplementary_tables
