# Supplementary material for: Diagnostic Performance of T2Candida Among ICU Patients With Risk Factors for Invasive Candidiasis
Source: Open Forum Infect Dis. 2019 Mar 25;6(5):ofz136. doi: 10.1093/ofid/ofz136 (PMC6501878; doi:10.1093/ofid/ofz136)
Supplement: ofz136_suppl_supplementary_tables [file ofz136_suppl_supplementary_tables.docx]

**Supplementary Tables**

**Table S-1.** Department characteristics for the two ICUs at the time of initiation of the study.

|  | Rigshospitalet ICU-4131  Copenhagen University Hospital | ICU-V  Odense University Hospital |
| --- | --- | --- |
| No. of beds | 23 | 27 |
| Bed days/year | 6944 | 8094 |
| Patients/year | 1323 | 2141 |
| % surgical patients | 48% | 43% |
| Age (median/IQR) | 59 (38-70) | 63 (39-73) |
| Mortality (ICU/day 30) | 11% / 17% | 11% / 20% |
| Apache II/SAPS II (median/IQR) | 23 (18-29) / 44 (33-58) | 24 (18-29) / 46 (35-56) |

**Table S-2.** Final classification and detailed clinical summary for each patient either with a first classification as proven, likely or possible, or with a positive T2Candida or MAg test result.

| **pt no** | **First classification** | **Final classificaion** | **Potential alternative Dx** | **Sex** | **Age** | **Primary diagnosis** | **Underlying diagnoses** | **Comment** | **Antifungal treatment before/at inclusion** | **Microbiology** | **Biomarkers** |
| --- | --- | --- | --- | --- | --- | --- | --- | --- | --- | --- | --- |
| 63 | proven | proven | yes | F | 48 | Intracerebral haemorrhage due to AV malformation. Perforated ulcus ventriculi. Stress induced cardiomyopathy | Laryngeal cancer. Opoid substitution therapy. Cerebral AV malformations | Ten days prior to inclusion the patient develops cerebral haemorrhage during an embolisation procedure for AV-malformation and secondary severe stress-induced cardiomyopathy with hypotension and multiple organ failure. On day of inclusion surgery (Billroth II) for perforated gastric ulcer | Fluconazole stopped the day before inclusion (T½=25-30 h) | *C. tropicalis* and *C. glabrata* in BC on date of inclusion; BC d 3 Neg. Colonised in respiratory tract with *C. albicans*, *C. tropicalis* and *C. glabrata* | T2: d0 C. alb./trop, d3: C. alb/trop & C. glab/krus Mannan Ag: >500 Mannan Ab: Intermediate |
| 103 | proven | proven | yes | M | 79 | neutropenic sepsis and multi-organ failure | Gastric cancer | On chemotherapy for gastric cancer. Admitted with neutropenic fever and septicaemia. Develops multi-organ failure. *C. tropicalis* in blood cultures. Improves on antibacterial and antifungal therapy. | Fluconazole <24h before inclusion | *C. tropicalis* in BC on date of inclusion and d2 and d4. Colonised with *C. tropicalis* in urine and respiratory tract. *Aspergillus fumigatus* in airway secretion d2, d4, d6, d9, d12, d13, d15 and d21 and in BAL d8 | T2: C. alb/trop Mannan Ag: >500 Mannan Ab: Neg |
| 4 | proven | Proven | yes | M | 71 | GI-bleeding | liver cirrhosis | Admitted with bleeding from oesophageal varices 10 days prior to study inclusion. Develops sepsis Growth of *C. glabrata* in blood cultures sampled the day before inclusion | no systemic (but topical nystatin) | *C. glabrata* in BC on the day of inclusion and day -1. *Staphylococcus epidermidis* in BC d-1 and d3 | T2: C. glab/krus Mannan Ag: Negative Mannan Ab: Intermediate |
| 98 | Proven | Proven | yes | F | 62 | Pneumonia | lung cancer, bladder cancer | Cystectomy 11 days before inclusion with construction of urethero-entero-cutaneostomy. Discharged 8 days after surgery but readmitted the next day with septic shock due to anastomosis leakage. During laparotomy the patient aspirates and develops severe circulatory collapse and respiratory failure. | none | *C. albicans* in BC also the day before inclusion. *Staphylococcus aureus* in BC, urine and pus from surgical site d-3. *Enterococcus faecalis* in urine d-3. Colonised with yeast in respiratory tract | T2: C. alb/trop Mannan Ag: Negative Mannan Ab: Negative |
| 33 | Proven | Proven | yes | M | 64 | GI-bleeding; aspiration pneumonia and MOF | Alcohol abuse, liver steatosis | Alcohol abuse, admitted with upper GI bleeding; aspiration pneumonia and sepsis with multiple organ failure (circulatory, pulmonary, renal, liver & CNS). *C. kefyr* in BC, most likely from CVC | anidulafungin 1d before inclusion | *C. kefyr* in BC at day of inclusion and 4 days later from CVK tip | T2: Negative Mannan Ag: >500 Mannan Ab: Intermediate |
| 89 | Proven | Proven | No | M | 71 | Hydronephrosis | Disseminated cardia cancer, hydronephrosis (JJ-catheters) | Admitted to hospital 15 days before inclusion due to poor general condition, dyspnoea and deteriorating kidney function. Is diagnosed with new right sided hydronephrosis day -6 and JJ-catheters are exchanged. The next day (d-5) a nephrostomy is performed. Deteriorates on d -2 with metabolic and respiratory acidosis and is transferred to ICU. Recovers on broad antibacterial and antifungal therapy. | none | *C. albicans* in BC 2 days before inclusion. Colonised with *C. albicans* in urine and respiratory tract | T2: C. alb/trop Mannan Ag: Negative Mannan Ab: Positive |
| 90 | Proven | Proven | yes | F | 85 | Colon transversum perforation, Faecal peritonitis | Endometrial cancer | Total hysterectomy for endometrial cancer. Two days later diagnosed with colon perforation and undergoes hemicolectomy the day before study inclusion. Growth of candida from swap from the abdominal cavity during surgery day 2. | Fluconazole 1d before inclusion | *Candida* from intraabdominal swap d2. *Serratia marcescens* in tracheal secretion at time of inclusion. *Enterococcus faecium*, *S. marcescens* in intraabdominal swap d2. | T2: C. glab/krus Mannan Ag: >500 Mannan Ab: Negative |
| 71 | Proven | Proven | yes | F | 61 | Necrotizing fasciitis secondary to perforated bowel | Ovary cancer - treated radically with surgery 6 years prior to inclusion | Day -12 laparoscopy for treatment of hernia. Day -5 discharge of faecal material from port hole. Day -3 admitted at local hospital. Develops necrotising fasciitis. Growth of candida albicans and 5 types of bacteria from tissue from abdominal wall | none | D -2 growth of *C. albicans*, *Citrobacter*, *Peptostreptococcus, Streptococcus anginosus, Prevotella* and *Fusobacterium* in sample of soft tissue from abdominal wall. Colonised with *C. glabrata* in urine. | T2: Negative Mannan Ag: Negative Mannan Ab: Intermediate |
| 119 | Proven | Proven | yes | M | 81 | Chronically infected aorta prosthesis | Abdominal aorta aneurism | 3 days before inclusion surgery with removal of infected prosthesis in abdominal aorta and closure of fistula between aorta graft and jejunum. | none | D -3 growth of *C. albicans*, *Staphylococcus epidermidis* and *E. faecium* in tissue from aorta and from aorta prosthesis. D9 *C. albicans* and *C. glabrata* in tissue from abdominal abscess cavity. D16 *C. albicans* from CVC tip | T2: Negative Mannan Ag: Negative Mannan Ab: Negative |
| 120 | Proven | Proven | yes | F | 61 | Mediastinitis, peritonitis | Diabetes, hypertension | Oesophageal tear 6-7 cm with secondary mediastinitis and peritonitis. The tear developed after 2 days with food jammed in eosophagus. Surgery x 2 the day of admission. At the second surgery (8.40 pm) drains are placed in mediastinum and peritoneum. Growth of Candida albicans from drains in mediastinum and peritoneum, samples drawn at 4 am the day after surgery (8 hours after placement of drains) | fluconazole | *C. albicans* and *Streptococcus mitis* d-2 and d1 from drains mediastinum and peritoneum <24h after drains are placed | T2: Negative Mannan Ag: Negative Mannan Ab: Negative |
| 16 | Proven | Proven | yes | M | 62 | sepsis and respiratory failure after cardia resection | Oesophageal cancer, diabetes | Undergoes cardia-resection for oesophageal cancer. Develops sepsis and respiratory failure. Growth of *C. glabrata* in pleural drain d2 after inclusion. The sample drawn the same day the drain is placed. | fluconazole <3d | d2 *C. glabrata* from drain in pleura, colonised with glabrata in respiratory secretion. *E. faecium* in gastric aspirate d3 | T2: Negative Mannan Ag: Negative Mannan Ab: Positive |
|  |  |  |  |  |  |  |  |  |  |  |  |
| 60 | likely | likely | yes | F | 67 | Necrotizing fasciitis and MOF | alcohol abuse, type 2 diabetes | Admitted to hospital 6 days prior to inclusion with necrotizing fasciitis. Treated with broad spectrum antibiotics. Develops multiple-organ failure. | Fluconazole initiated on day of inclusion | Group G *Streptococcus* in bone from femur (2 samples) d-5. Colonised with *C. albicans* in respiratory tract and with *C. krusei* in respiratory tract and urine. *C. dubliensis* in BC 5 days after inclusion and *Mucor* in tracheal 5 days after inclusion. | T2: C. glab/krus Mannan Ag: Positive Mannan Ab: Positive |
| 22 | likely | likely | yes | F | 68 | Peritonitis, haemorrhagic diathesis, respiratory failure | Disseminated adenocarcinoma (unknown primary tumour, but GI tract) | Upper GI-bleeding and haemorrhagic diathesis. CT raises suspicion of perforated bowel, which is not confirmed at explorative laparotomy, but there is an abscess around appendix. Autopsy shows metastases in bone marrow, lungs, large bowel and spleen. Initial classification: likely due to colonization and no alternative microbiological diagnosis. Revised diagnosis: likely due to same reasons + *C. tropicalis* at CVC tip 5 days after inclusion | Fluconazole 4d before inclusion | Colonised with *C. tropicalis* in urine and respiratory tract. *C. tropicalis* and S. epidermidis at CVC tip 5 days after inclusion and in *C. tropicalis* in peritoneum day 9 | T2: C. alb/trop Mannan Ag: Positive Mannan Ab: Positive |
| 28 | possible | likely | No | F | 25 | Sepsis and (Sec) pulmonary infection and respiratory failure | Hodgkin Lymphoma | Respiratory failure and treated with ECMO. At bronchoscopy the lower airways are covered with thrush. BAL with candida albicans 10^4/ml. Initial classification: possible due to colonization, but alternative microbiological diagnosis. Revised classification: Likely due to the findings at bronchoscopy and heavy growth of candida in BAL. | Fluconazole | *C. albicans* ++ faeces, yeast gastric asp 5/2, *C. albicans* ++ trach asp, yeast +++ faeces, *C. albicans* ++ BAL -->10(4)/ml in BAL 15/2. then (d6) also BAL GM pos (6.8) but serum neg. and BAL *Aspergillus* GM at incl. (0.2). EBV 14,000 copies/ml in blood d1, | T2: C. alb/trop Mannan Ag: Positive Mannan Ab: Negative |
| 116 | possible | likely (proven d-21 and d-5) | yes | F | 81 | Colovesical fistel, continuous bladder perforation to peritoneum | polymyalgia (on corticosteroids), diabetes | Develops colovesical fistula 6 weeks before inclusion, first treated conservatively. Surgery with colostomy and closure of fistula 3 weeks before inclusion. At time of surgery *C. tropicalis* and *C. glabrata* in abdominal drain. Treated with fluconazole. There is continuously defects (sized 2 cm) in bladder wall with communication to peritoneum. Five days before inclusion cystoscopy still shows defects as well as food items and faecal matter in the bladder and a new abdominal drain is placed from which there is growth of *C. tropicalis* and *C. glabrata* (sample from same day). There is more urine output from abdominal drain than from bladder catheter. D-1 *C. tropicalis* and *C. glabrata* in urine. The patient improves clinically after switch from fluconazole to anidulafungin. Initial classification: possible due to colonization and sepsis but also growth of *E. faecium*. Revised classification: likely because fulfilment of criteria for proven invasive infection d-5 and because there is communication between bladder and peritoneum and *C. glabrata* and *C. tropicalis* in urine d-1. The sample from the abdominal drain that day could not be analysed in the laboratory. The patient improves after switch to anidulafungin. | Fluconazole >7d before inclusion, switch to anidulafungin at inclusion | *C. tropicalis* and *C. glabrata* in abdominal drain d-21 (drain placed the same day), *C. tropicalis* and *C. glabrata* in abdominal drain d-5 (drain placed the same day) *C. glabrata* in abdominal drain d-2 (drain not new), *C. tropicalis* and *C. glabrata* in urine d-1. Sample from abdominal drain d-1 could not be analysed. No growth of bacteria other than *E. faecium* from aspirate from ventricle (stomach) d-1. Colonised with yeast in respiratory tract secretion. | T2: Negative Mannan Ag: Negative Mannan Ab: Positive |
| 54 | possible | likely (proven d-4) | yes | M | 43 | Peritonitis | Neuroendocrine tumour | Ten days before inclusion the patient undergoes total pancreadectomy with ileocoecal resection and splenectomy due to neuroendrocrine tumour. On day -4 an exploratory laparotomy is performed due to sepsis. Findings: perforation of large bowel and biliary tract. Initial classification: Possible due to colonization and sepsis, but alternative microbiological diagnosis. Revised classification: Likely due to proven invasive *C. albicans* infection 4 days before inclusion and growth of *C. albicans* in several samples from drains in the abdomen | Fluconazole 4d before inclusion | d-5 *Pseudomonas* and *C. albicans* from drain in abdomen d-4 *C. albicans* in peritoneum (per-operative) d-3 *C. albicans* from drain in gall bladder d-3 *C. albicans* + *E .faecium* from drain in gall bladder d0 *E. faecium* and *Micrococcus luteus* from drain in abdomen d+6 *E. faecium* and *C. albicans* from abscess in abdomen | T2: C. alb/trop Mannan Ag: Negative Mannan Ab: Intermediate |
| 47 | likely | likely (proven  d-4) | yes | F | 71 | Necrotizing fasciitis secondary to perforated appendicitis. Intraabdominal abscess. Bowel ischaemia | None | Septic at admission 12 d prior to inclusion. CT abdomen shows perforated appendicitis and abscess that extends to soft tissues at abdomen. Immediate surgery with findings of torquation of small bowel with necrotic ileum and perforation in coecum. Necrotizing fasciitis of abdominal wall from os pubis up to sternum. Initial classification: possible due to colonization, but possible alternative microbiological diagnosis. Revised classification: Likely. There is *C. albicans* in newly placed abdominal drain 4d prior to inclusion and d0 in a drain placed >1d before sampling. Also *C. dubliniensis* in abdominal drain d10. | Fluconazole | d-12: *Aggregatibacter*, *E. coli*, *B. fragilis* & *Streptococcus anginosus* from soft tissue (abdomen) d-12: *Streptococcus anginosus* in pus from intraabdominal abscess d-12: G- rods in swab from subcutaneous tissue (abdomen) d-4: *C. albicans* in drain from inner abdomen (drain placed exactly 24 h before sampling) d0: *C. albicans* in drain from inner abdomen (drain placed >1d before sampling) and *C. albicans* in respiratory secretion d10: *C. dubliniensis* in drain from inner abdomen | T2: Negative Mannan Ag: Negative Mannan Ab: Positive |
|  |  |  |  |  |  |  |  |  |  |  |  |
| 121 | unlikely | possible (proven d4) | yes | M | 84 | Post-surgery bowel perforation and peritonitis | gastric cancer, ischemic heart disease, nephropathy | Previously gastrectomy due to cancer. Develops two stenosis. 4 days prior to inclusion surgery with dilatation and revision of stenosis. At day of inclusion explorative laparotomy which reveals leakage in upper small intestine with severe inflammation and peritonitis. Growth of 3 types of GI bacteria from drain. Colonised with yeast in respiratory tract. There is growth of yeast in swap from peritoneum collected at a secondary laparotomy d4 after inclusion. Initial classification: Unlikely - only colonised at one site and alternative microbiological diagnosis. Revised classification: Possible. Yeast is found in samples from abdomen day 4 after inclusion but bacterial infection is a likely cause of sepsis. | Fluconazole | D1 *E. faecium*, *Enterobacter* and *K. pneumoniae* from abdominal drain D4 swap (per-operatively) from peritoneum with yeast D6 weak growth of *C. albicans* from abdominal drain >24h after placement of drain Colonised with *C. albicans* in respiratory tract | T2: Negative Mannan Ag: >500 Mannan Ab: Positive |
| 20 | unlikely | Possible (proven  d-7 and -5) | yes | F | 65 | Pancreatitis and peritonitis secondary to perforated gastric ulcer | Alcohol abuse | Admitted to hospital 11 days prior to study inclusion with acute pancreatitis. Three days later complicated with perforated gastric ulcer and peritonitis. Several complications during the following weeks. Re-perforation of the stomach and of the gall-bladder. Abdominal drains placed on several occasions. After weeks with septic shock and multiple organ failure, active treatment is stopped and the patient dies. Initial classification: Unlikely since there are no findings of candida other than from a sample of tracheal secretion within 3 days of study inclusion. Revised classification: possible because there is growth of *C. albicans* in blood cultures 7 and 5 days before inclusion and the patient is on antifungal treatment prior to inclusion. | Fluconazole d -7 to d -3 and Caspofungin from d -3 | *C. albicans* in blood culture d-7, d-5 and abdominal drain d9. Colonised with *C. albicans* in respiratory tract. Coagulase negative Staph from CVC tip d1 and from peritoneum d4, d5, d7 and blood d7. | T2: Negative Mannan Ag: Negative Mannan Ab: Positive |
| 52 | likely | Possible, (Proven  d -7) | yes | F | 61 | Septic shock, focus in urinary tract | Chronic nephropathy and JJ-catheters. Alcohol abuse | Septic, anuric with bilateral hydronephrosis. Nephrostomy is performed 7d before inclusion. *C. albicans* and *Klebsiella* *pneumoniae* in pus from the nephrostomy. Initial classification: likely because colonised and septic and no alternative microbiological diagnosis within 3 days of inclusion. Revised classification: possible due to proven invasive candida 7d before inclusion and started antifungal treatment prior to inclusion. Potentially bacterial infection as cause of septicaemia. | Fluconazole | *C. albicans* and *Klebsiella pneumoniae* in pus from the nephrostomy 7d before inclusion. Colonised with *C. albicans* in catheter-urine and respiratory tract at time of inclusion. ('10(5) C. alb KAD urine, +++C. alb kidney drain). *Clostridium difficile* 2d after inclusion | T2: Negative Mannan Ag: Negative Mannan Ab: Positive |
| 43 | likely | possible | yes | F | 88 | Peritonitis secondary to ischaemic small bowel and bowel resection | hypertension | Admitted to hospital 17 days prior to inclusion due to abdominal pain. Surgery 5 days later with findings of ischemic terminal jejunum and peritonitis. Undergoes bowel resection with side to side anastomosis. Abdominal fascia rupture 5 days prior to inclusion and undergoes surgery again. Has septic shock and is transferred to ICU 2 days prior to inclusion. Initial classification: Likely due to colonization at two sites and no other positive microbiological findings within 3 days. Revised classification: Possible due to colonization and sepsis, but bacterial infection is a likely alternative diagnosis despite negative blood and urine cultures. | Fluconazole 2d before inclusion | Colonised with *C. albicans* in catheter-urine and respiratory tract | T2: Negative Mannan Ag: Negative Mannan Ab: Positive |
| 48 | likely | possible | yes | M | 49 | peritonitis secondary to incarcerated hernia and small bowel resection | alcohol abuse and liver cirrhosis | Admitted to hospital 6 days prior to inclusion due to abdominal pain. Diagnosed with incarcerated inguinal hernia and undergoes small bowel resection due to ischemic bowel. Subsequently he develops peritonitis. Initial classification: Likely due to colonization at two sites and no other positive microbiological samples within 3 days. Revised classification: Possible due to colonization and sepsis, but bacterial infection is a likely alternative diagnosis due to resent necrotic bowel with resection and secondary peritonitis. | fluconazole started day of inclusion | Colonised with *C. albicans* in catheter-urine and respiratory tract, 10(4-5) *C. albicans* KAD urine, +++ *C. albicans* in tracheal aspirate | T2: Negative Mannan Ag: Negative Mannan Ab: Positive |
| 114 | possible | possible | yes | M | 57 | Pneumonia, Perforated peptic ulcer | alcohol abuse, COPD | Admitted to hospital 4 days before inclusion with a primary diagnosis of pneumonia and respiratory failure. Develops perforated ulcers of the upper GI tract including the oesophagus. Severe peritonitis and septic shock with multiple organ failure. Develops several intraabdominal abscesses, which are drained. Growth of candida in samples from abdominal drains 6 days after study inclusion. | Fluconazole | Colonised with *C. albicans* in respiratory tract. *C. albicans* in abdominal drains from day 6 to day 19 (9 samples). *E. faecium* in abdominal drains from day 6 to day 13 (4 samples) and in BC day 22 | T2: C. alb/trop Mannan Ag: Negative Mannan Ab: Intermediate |
| 58 | possible | possible | yes | M | 65 | Pancreatitis secondary to bile stones. MOF | None (alcohol abuse) | Pancreatitis and intraabdominal abscesses. Sepsis and multiple organ failure. Improves with placement of drains, antibacterial treatment and fluconazole. Deteriorates after drains are displaced and fluconazole is discontinued. Initial classification: possible due to colonization, sepsis and potentially bacterial infection as cause of sepsis. Revised classification: possible for same reasons | None | Colonised with yeast in respiratory tract and urine. Colonised with *E. faecium* in urine.  D12 *C. glabrata* in drain from abdominal abscesses | T2: Negative Mannan Ag: Negative Mannan Ab: Negative |
| 59 | possible | possible | yes | M | 67 | GI-bleeding, repeated abd.surgery, biliroth II | diabetes, alcohol abuse, neuroendocrine tumour | Repeated GI-bleeding. Surgery x 2 to achieve haemostasis. Biopsies from surgery diagnostic for neuroendocrine tumour. *C. albicans*, *C. tropicalis* and *C. glabrata* from drains from abdominal abscesses, but sampling >1 d after drains are placed. First classification: Possible because colonised, septic and potentially bacterial infection as cause of sepsis. Revised classification: Possible for same reasons | Fluconazole | Colonised with yeast in respiratory tract d-2 *C. albicans*, *C. tropicalis* and *C. glabrata* from abdominal drain (right) (drain placed 3d prior to sampling) d-2 *C. albicans* and *C. tropicalis* from abd. drain (left) (drain placed 3d prior to sampling) d0 *Clostridium difficile* d3 *C. albicans*, *C. tropicalis* and *C. glabrata* from abd. drain (right) d3 *C. albicans* and *C. tropicalis* from abd. drain (left) d14 *C. glabrata*, *E. faecium* and *Lactobacillus* from drain in gall bladder | T2: Negative Mannan Ag: Negative Mannan Ab: Positive |
| 100 | possible | possible | yes | F | 68 | intraabdominal infection secondary to leakage after whipple surgery | pancreatic cancer, cutaneous T-cell lymphoma, diabetes | Surgery (whipple) 23d before inclusion. Leakage from hepatico-jejunostomy. Multibacterial sepsis. Intra-abdominal abscesses. Initial classification: Possible due to colonization, sepsis and alternative microbiological diagnosis. Revised classification: possible for the same reasons. There is repeated finding of *C. parapsilosis* from abdominal drain, but the drain is placed >1d before sampling | Fluconazole 2d before inclusion | *Pseudomonas, Serratia, E. faecalis, E. faecium, Enterobacter cloacae, S. aureus* and *S. haemolyticus* found in abdominal drain/blood during the admission from September to December. *C. parapsilosis* from abdominal drain d-4, d-1 and d6 and also in urine. *C. albicans* in urine. | T2: Negative Mannan Ag: Negative Mannan Ab: Negative |
| 70 | unlikely | possible | yes | M | 69 | Perforated gastric ulcer and peritonitis | Chronic pancreatitis, type 2 diabetes | Undergoes gastocystotomia for pancreatic cyst 7 days prior to inclusion. Re-admitted the day before inclusion where laparotomy is performed with findings of severe peritonitis. Develops septic shock and multiple organ failure. Initial classification: unlikely because there are no samples with growth of candida within 3 days of inclusion. Revised classification: Possible due to growth of c albicans from abdominal drain 8 days after inclusion in a patient with septicemia and perforation of GI-tract. | Fluconazole initiated on day of inclusion | No colonization. *C. albicans*, *E. faecium*, *E. coli* and *Enterobacter cloacae* from abdominal drain d8 after inclusion | T2: Negative Mannan Ag: Positive Mannan Ab: Negative |
| 74 | unlikely | possible | yes | M | 78 | Faecal peritonitis secondary to perforated diverticulitis | Disseminated esophageal cancer | Esophageal cancer. Chemotherapi with the aim of downstaging before oesophageal surgery. Admitted to hospital 8 days prior to inclusion due to abdominal pain and sepsis. The day before inclusion laparatomy is performed with finding of perforated diverticullitis. Initial classification: unlikely because there is only documented colonization in one site. Revised classification: possible due to septicaemia despite broad spectrum antibacterial treatment in a patient who has been treated with chemotherapy and has perforation of the GI-tract and heavy yeast colonization of the respiratory tract. | fluconazole 1d before inclusion | Colonised with *C. albicans* and *C. tropicalis* in respiratory tract (+++). *A. fumigatus* in sputum d6 | T2: Negative (C. glab/krus d4) Mannan Ag: Negative (Positive, 125, d4) Mannan Ab: Negative |
|  |  |  |  |  |  |  |  |  |  |  |  |
| 83 | unlikely | unlikely | yes | M | 67 | bacteraemia (Sreptococcus), meningitis | None | Streptococcal bacteremia with meningitis and multiple organ failure. Initial classification: Unlikely since there is no growth of candida in microbiological samples. Revised classification: Unlikely for the same reason. | fluconazole | Gr. A *Streptococcus* in blood d-5. *Erwinia* in CSF d-1. Not colonised with yeast | T2: C. para d1 Mannan Ag: Negative Mannan Ab: Negative |
| 109 | possible | unlikely | yes | F | 53 | CMV, hemophagocytosis | SLE | SLE and immunosuppressive therapy. Reactivation of CMV and secondary hemophagocytic syndrome. Treated with etoposide and dexamethasone. Primary classification: possible due to sepsis, colonization with yeast and other microbiological diagnosis. Reclassification: Unlikely: There is nothing that indicates invasive fungal infection and there is an alternative diagnosis for the septic condition. | None | Colonised with yeast in urine and respiratory tract. CMV PCR in blood positive 55,000 copies/ml | T2: Negative Mannan Ag: >500 Mannan Ab: Negative |
| 24 | unlikely | unlikely | yes | M | 75 | Aspiration pneumonia | pulmonary cancer (no chemotherapy) | Lobectomy for pulmonary cancer 7 weeks prior to inclusion. Admitted to the ICU the following 25 days. Discharged from ICU, but readmitted to the ICU 10 days later due to aspiration pneumonia. | Fluconazole | *K. pneumonia* in respiratory tract d-9. *Stenotrophomonas* in respiratory tract d-4, d-2 and d0.Serratia in pleural drain d-3, in respiratory tract d7 and in blood culture d10, *Corynebacterium* and yeast in pleural drain d-3, *E. faecium* in urine d0. Colonised with *C. albicans* in respiratory tract | T2: Negative Mannan Ag: Positive (175) Mannan Ab: Positive |
| 91 | possible | unlikely | yes | F | 79 | Pneumonia | Polycytemia vera, COPD | Admitted with respiratory tract infection. Initial improvement on antibiotics, but suddenly develops sepsis. Colonised with yeast in urine and respiratory tract. Initial classification: possible due to sepsis and colonization but also alternative microbiological diagnosis. Revised classification: Unlikely, since there is no indication of invasive candidiasis | None | Colonised with yeast in urine and respiratory tract. Mould in respiratory secretion d-1 | T2: Negative Mannan Ag: Negative Mannan Ab: Positive |
| 112 | possible | unlikely | yes | F | 30 | pancreatitis | alcohol abuse, diabetes | Admitted with pancreatitis and metabolic acidosis. Acinobacter in blood culture 4 d before inclusion. Colonised with *C. albicans* in urine and respiratory secretions, but no evidence of invasive candidiasis. Initial classification: possible due to colonization, sepsis and alternative microbiological diagnosis. Revised classification: unlikely since there is no indication of invasive candidiasis | Fluconazole | *Acinetobacter* in blood culture d-4. *C. albicans* in urine (10^4/ml) and respiratory secretions | T2: Negative Mannan Ag: Negative Mannan Ab: Positive |
| 36 | possible | unlikely | yes | F | 49 | Influenza A, secondary bacterial pneumonia | Diabetes, alcohol abuse, Chronic pancreatitis | Severe influenza A (H1N1). Develop secondary staph aureus pneumonia and bacteremia. Also staph epidermidis in blood and tip of CVC. Intial categorization: possible due to sepsis and colonization with *C. albicans* in urine and respiratory tract but also alternative microbiological diagnosis. Revised classification: unlikely, since there is an alternative diagnosis and no evidens for invasive candidiasis. | Fluconazole | Influenza A (H1N1) in respiratory tract samples from 8 days before to 5 days after inclusion. Coagulase negative staph in blood from 3 days before to 6 days after inclusion as well as from CVC tip 7 days after inclusion. *S. aureus* in respiratory tract samples and in blood 5 and 10 days after inclusion. *C. albicans* in urine and respiratory tract samples | T2: Negative Mannan Ag: Negative Mannan Ab: Negative |
| 57 | possible | unlikely | yes | F | 46 | Sepsis with suspected pulmonary focus | Tonsil cancer disseminated to regional lymph nodes | Deterioration over 2 months. 7d prior to inclusion diagnosed with tonsil cancer. Septic with suspected pulmonary focus. BAL 1d prior to inclusion positive for influenza B. Initial classification: possible due to colonization and sepsis, but alternative microbiological diagnosis. Revised classification: Unlikely due to clinical picture of respiratory tract infection compatible with severe influenza and no signs of invasive candidiasis | Caspofungin and prior to that fluconazole. Fungizone for bladder irrigation | *C. krusei* in urine and secretion from respiratory tract. BAL d-1 PCR positive for influenza B | T2: Negative Mannan Ag: Negative Mannan Ab: Negative |
| 80 | possible | unlikely | yes | F | 79 | Hip infection (prosthesis) with MRSA | Diabetes | One 1 month after placement of a hip prosthesis the patient develops hip infection with MRSA. The prosthesis is removed. Secondary revision of the hip after a month. Develops septic shock, VAP and multiple organ failure after the revision and is included in the study at that time. Initial classification: possible due to colonization and alternative microbiological diagnosis. Revised classification: Unlikely, Has hip infection with MRSA and VAP after surgery. No evidence of invasive fungal infection | Caspofungin <24h prior to inclusion | Colonised with *C. glabrata* in urine and respiratory tract at incl. MRSA from tissue from hip 30 days prior to inclusion. No growth in 4 of 4 tissue samples, pus from femur or from foreign body d-1 | T2: Negative Mannan Ag: Negative Mannan Ab: Negative |
